# High platelet reactivity strongly predicts early stent thrombosis in patients with drug-eluting stent implantation

**DOI:** 10.1038/s41598-023-50920-9

**Published:** 2024-01-04

**Authors:** Subin Lim, Soon Jun Hong, Ju Hyeon Kim, Jung-Joon Cha, Hyung Joon Joo, Jae Hyoung Park, Cheol Woong Yu, Byeong-Keuk Kim, Kiyuk Chang, Yongwhi Park, Young Bin Song, Sung Gyun Ahn, Jung-Won Suh, Sang Yeub Lee, Jung Rae Cho, Ae-Young Her, Young-Hoon Jeong, Hyo-Soo Kim, Moo Hyun Kim, Eun-Seok Shin, Do-Sun Lim, PTRG Investigators, Hyun Kuk Kim, Jung Hee Lee, Byoung Kwon Lee, Weon Kim, Kyung Woo Park, Jae Yeon Moon, Osung Kwon, Chan Joon Kim, Hyun-Woong Park, Chang Hoon Lee, Woo Jin Jang, Han-Young Jin, Min Ku Chon, Ki Hong Choi, Dong Hoon Han, Min Gyu Kang, Jeehoon Kang, You Jeong Ki, Jin Sup Park, Seung-Jun Lee, Seung Hun Lee, Jong-Young Lee, Sung Won Cho, Jon Suh, Jang-Whan Bae, Seng Chan You, Do-Sun Lim, Myung Ho Jeong

**Affiliations:** 1 Department of Cardiology, Cardiovascular Center, Korea University Anam Hospital, Korea University College of Medicine, 73, Goryeodae-ro, Seongbuk-gu, Seoul, 02841 Republic of Korea; 2 Severance Cardiovascular Hospital, Seoul, Republic of Korea; 3 Division of Cardiology, Department of Internal Medicine, College of Medicine, Catholic University of Korea, Seoul, Republic of Korea; 4 Department of Internal Medicine, Gyeongsang National University School of Medicine and Cardiovascular Center, Gyeongsang National University Changwon Hospital, Changwon, Republic of Korea; 5 Division of Cardiology, Department of Medicine, Samsung Medical Center, Sungkyunkwan University School of Medicine, Seoul, Republic of Korea; 6 Department of Cardiology, Yonsei University Wonju Severance Christian Hospital, Wonju, Republic of Korea; 7 Department of Internal Medicine, Seoul National University College of Medicine and Department of Cardiology, Seoul National University Bundang Hospital, Seongnam, Republic of Korea; 8 Department of Cardiology, Chung Ang University Gwangmyeong Hospital, Gwangmyeong, Republic of Korea; 9 Cardiology Division, Department of Internal Medicine, Kangnam Sacred Heart Hospital, Hallym University College of Medicine, Seoul, Republic of Korea; 10 Division of Cardiology, Department of Internal Medicine, Kangwon National University School of Medicine, Chuncheon, Republic of Korea; 11 Department of Internal Medicine and Cardiovascular Center, Seoul National University Hospital, Seoul, Republic of Korea; 12 Department of Cardiology, Dong-A University Hospital, Busan, Republic of Korea; 13 Division of Cardiology, Ulsan University Hospital, University of Ulsan College of Medicine, Ulsan, Republic of Korea; 14 Department of Internal Medicine and Cardiovascular Center, Chosun University Hospital, Gwangju, Republic of Korea; 15 Cardiology Division, Gangnam Severance Hospital, Seoul, Republic of Korea; 16 Department of Cardiology, Kyung Hee University Hospital, Seoul, Republic of Korea; 17 Department of Cardiology, CHA Bundang Medical Center, CHA University, Seongnam, Republic of Korea; 18 Division of Cardiology, Department of Internal Medicine, Eunpyeong St. Mary’s Hospital, College of Medicine, The Catholic University of Korea, Seoul, Republic of Korea; 19 Department of Internal Medicine, Division of Cardiology, Uijeongbu St Mary’s Hospital, College of Medicine, The Catholic University of Korea, Seoul, Republic of Korea; 20 Department of Cardiology, Veterans Health Service Medical Center, Seoul, Republic of Korea; 21 Department of Cardiology, Ewha Womans University, Seoul, Republic of Korea; 22 Cardiovascular Center, Inje University Busan Paik Hospital, Busan, Republic of Korea; 23 Division of Cardiology, Pusan National University Yangsan Hospital, Yangsan, Republic of Korea; 24 Department of Cardiology, Uijeongbu Eulji Medical Center, Eulji University, Uijeongbu, Republic of Korea; 25 Department of Cardiology, Pusan National University Hospital, Busan, Republic of Korea; 26 Division of Cardiology, Chonnam National University Hospital, Gwangju, Republic of Korea; 27 Division of Cardiology, Kangbuk Samsung Hospital, Sungkyunkwan University College of Medicine, Seoul, Republic of Korea; 28 Division of Cardiology, Cheju Halla General Hospital, Jeju, Republic of Korea; 29 Department of Cardiology, SoonChunHyang University Bucheon Hospital, Bucheon, Republic of Korea; 30 Division of Cardiology, Department of Internal Medicine, Chungbuk National University College of Medicine, Cheongju, Republic of Korea

**Keywords:** Interventional cardiology, Cardiology

## Abstract

Stent thrombosis (ST) is a fatal complication after percutaneous coronary intervention (PCI). The association between P2Y12 reaction unit (PRU) level and stent thrombosis occurrence remains unclear. Based on the multicenter, observational PTRG-DES (Platelet function and genoType-Related long-term proGnosis in DES-treated patients) registry of patients with drug-eluting stents (DES) implantation, a total of 11,714 patients with PRU values were analyzed. We sought to identify the predictors of early stent thrombosis (EST) and compared the primary outcome, a composite of cardiac death, myocardial infarction, and revascularization, between EST and non-EST groups. EST, defined as definite ST within 1 month after index PCI, occurred in 51 patients. PRU values were significantly higher in the EST group (263.5 ± 70.8 vs. 217.5 ± 78.7, *p* < 0.001). In multivariable analysis, PRU ≥ 252 (OR, 5.10; 95% CI 1.58–16.46; *p* = 0.006) and aspirin reaction unit ≥ 414 (OR 4.85; 95% CI 1.07–21.97; *p* = 0.040) were independent predictors of EST. The cumulative incidence of primary composite outcome at one year was significantly higher in the EST group (38.2% vs. 3.9%, Log-rank *p* < 0.001). In patients treated with clopidogrel after successful DES implantation, EST was associated with higher platelet reactivities, and a greater risk of cardiovascular events.

Trial Registration: clinicaltrials.gov Identifier: NCT04734028.

## Introduction

Percutaneous coronary intervention (PCI) with drug-eluting stent (DES) implantation is an effective treatment modality for coronary artery disease (CAD), with significant cardiovascular benefits. Despite these benefits, early stent thrombosis (EST) remains a rare but potentially devastating complication of DES implantation, with high rates of morbidity and mortality. EST, frequently presenting as acute coronary syndrome (ACS), can lead to additional myocardial infarction (MI) and sudden cardiac death after its occurrence, even after its resolution^1–3^. Platelet reactivity often hinders the activity of antiplatelet drugs and is a risk factor for EST, but its use is currently limited in pragmatic clinical settings.

Current guidelines support the use of dual antiplatelet therapy (DAPT) with a P2Y12 inhibitor and aspirin after PCI to prevent ST. Clopidogrel, however, is a prodrug dependent on hepatic metabolism for activation, which makes it susceptible to individual variations in potency, and sometimes inactivity as a result^4,5^. Nevertheless, routine testing of platelet reactivity or gene testing is not currently recommended in clinical practice.

In this study, the authors sought to investigate the association between high platelet reactivity (HPR) and the risk of EST, as well as the clinical outcomes after EST, in a large cohort of patients undergoing PCI with DES implantation.

## Methods

### Study population

The Platelet function and genoType-Related long-term proGnosis in DES-treated patients (PTRG-DES) registry is the cumulation of nine prospective registries of patients who were treated with DES from 32 Korean academic centers^6^. All consecutive patients treated with one or more DES during PCI were screened for enrolment. Patients who underwent DES PCI and received adequate loading and maintenance doses of dual antiplatelet therapy (DAPT) with aspirin and clopidogrel were eligible.

**Figure 1:**
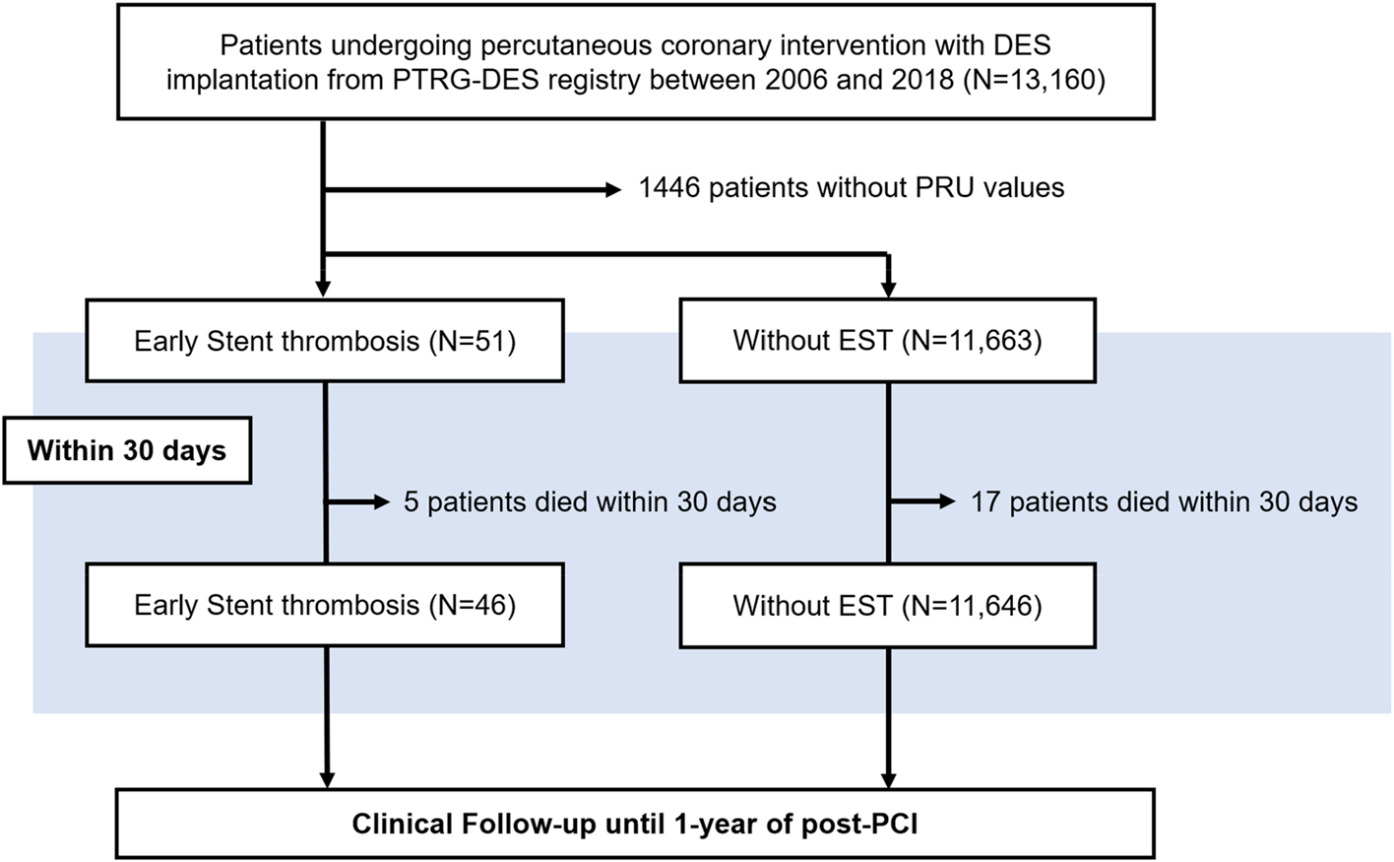
Study flowchart.

Between July 2003 and August 2018, 13,160 patients were enrolled, among which 11,714 patients with platelet function test (PFT) measurement values (as measured by VerifyNow assay) were identified (Fig. 1). The major exclusion criteria were: (1) the occurrence of a major complication during the procedure; (2) the use of fibrinolytic therapy; (3) any need for oral anticoagulation or potent P2Y12 inhibitor (such as ticagrelor or prasugrel); or (4) PCI strategy other than DES.

Follow-up was conducted through visits to the outpatient clinic or telephone calls during each study period. The follow-up data in this consortium were subsequently updated by analyzing the electronic medical records. All clinical events were evaluated and adjudicated by an independent committee who were masked to the study results. The follow-up was censored upon the occurrence of the first clinical event or the discontinuation of clopidogrel.

### Procedures

All patients were treated with DES during PCI and were adequately given adequate loading doses of aspirin and clopidogrel prior to enrolment, which was as follows: aspirin was given as either (1) a coated oral dose of 300 mg at least 6 h or (2) a dose of 100 mg at least 5 days before PCI. Clopidogrel was given as either (1) a dose of 600 mg at least 6 h, (2) a dose of 300 mg at least 12 h, or (3) a dose of 75 mg at least 5 days before PCI. Following PCI, all patients were administered 100 mg of aspirin and 75 mg of clopidogrel for maintenance doses. Aspirin was maintained indefinitely and clopidogrel was given for at least 1 year following index PCI. Patients with other compelling indications for oral anticoagulation, potent P2Y12 inhibitors (ticagrelor or prasugrel) or abciximab (due to its long washout period) were excluded from the study.

All PCI procedures were conducted according to standard protocols, and all other treatments were per standard of care. Clinical outcomes were evaluated until the last outpatient visit. All clinical events were adjudicated by an independent clinical events committee masked to VerifyNow results.

### Platelet function tests

The VerifyNow assay (Accriva, San Diego, CA, USA) was used to measure platelet reactivity. VerifyNow P2Y12 baseline reactivity, P2Y12 reaction units (PRU) and VerifyNow aspirin reaction units (ARU) values were collected for the study. All platelet reactivity values were measured after PCI, after being given loading doses of aspirin and clopidogrel, and within 24 h after DES implantation.

The VerifyNow assay is a whole-blood, point-of-care, turbidimetric optical detection assay designed to measure agonist-induced platelet aggregation. Blood samples were collected in 3.2% citrate Vacuette tubes (Greiner Bio-One Vacuette North America, Monroe, NC, USA). The measurement protocol followed the manufacturer’s recommendations. The details of the measurement protocol are described elsewhere^7^.

### Clinical outcome definitions

The primary outcome was the occurrence of a composite outcome of cardiac death, myocardial infarction (MI) and any revascularization between 30 days and 1 year since the index date. Key secondary outcomes were all-cause death, cardiac death, MI and any revascularization from after 30 days since the index date.

Cardiac death was defined as all deaths due to MI, cardiac tamponade, fatal arrhythmia, or related to procedural complications (as adjudicated after the procedure). All other deaths were considered cardiac deaths unless a definite non-cardiac cause could be established. MI was defined as an increase in serum cardiac markers (either creatinine kinase-myocardial band (CK-MB), troponin T or troponin I) greater than the 99th percentile of the upper limit of normal combined with the presence of clinical evidence of MI (symptoms, electrocardiographic changes or abnormal imaging findings associated with MI). Peri-procedural MI, or MI associated with the procedure were excluded from MI diagnosis. ST was defined as definite ST according to the Academic Research Consortium criteria^8^. Early ST was defined as definite ST occurring within 1 month of the index procedure. Any revascularization included bypass surgery or PCI on either target or nontarget vessels, except for the revascularization for EST in the EST group.

### Statistical analysis

Categorical variables are reported as counts and percentages and were compared between groups using the chi-square test or Fisher’s exact test as appropriate. Continuous variables are reported as mean ± SD and were compared between groups using Student’s t-test or the Mann–Whitney U test as appropriate. The cumulative incidences of the primary (the composite of cardiac death, MI, and any revascularization) and secondary (all-cause death, cardiac death, MI, and any revascularization) outcome events are presented as Kaplan–Meier estimates and were compared between groups using the log-rank test. Univariable and multivariable logistic regression analyses were performed to compute odds ratios (OR) with 95% confidence intervals (CI). For the multivariable model, variables with a *p* value of < 0.20 and major clinical risk factors were included for analysis, which were as follows: sex, old age (≥ 75 years), left ventricular ejection fraction (LVEF), diabetes mellitus, dyslipidemia, hypertension, prior stroke history, anemia, multivessel disease, bifurcation lesion, PCI at left main (LM) or left anterior descending artery (LAD), PRU ≥ 252 and ARU ≥ 414. The cut-off values of PRU and ARU were determined based on previous literature^9^.

A *p* value of < 0.05 was considered to be statistically significant. Statistical analyses were performed using R statistical software (version 4.1.2; R Foundation for Statistical Computing, Vienna, Austria).

## Results

### Patient and procedural characteristics

A total of 13,160 patients undergoing PCI with DES implantation were enrolled between July 2003 and August 2018 (Fig. 1). After the exclusion of 1446 patients without PRU values, a final cohort of 11,714 patients was selected for analysis. Of these, 51 (4.3%) patients had EST.

Baseline clinical characteristics according to the occurrence of EST are summarized in Table 1. The average age was 64.4 ± 10.9 years and 8848 (67.2%) were men. The two groups did not differ significantly in terms of baseline demographics including age, sex, and major clinical risk factors such as hypertension, dyslipidemia, smoking status, diabetes mellitus, chronic kidney disease, anemia and presentation as AMI. The glomerular filtration rate was lower in the EST group. There were no significant differences between the two groups in terms of lipid profiles. By CYP2C19 genotyping, a higher percentage of patients in the EST group were intermediate (14 (56.0%) vs. 3217 (48.1%)) and poor (8 (32.0%) vs. 949 (14.2%)) metabolizers. (Supplemental Table S1) On the contrary, a higher percentage of patients without EST were extensive metabolizers compared to those with EST (2526 (37.7%) vs. 3 (12.0%)).

**Table 1:** Baseline characteristics of patients according to the occurrence of early stent thrombosis. (PTRG-PFT).

	Overall (n = 11,714)	Early stent thrombosis (−) (n = 11,663)	Early stent thrombosis (+) (n = 51)	*p* value
Presentation as AMI	3338 (28.5%)	3318 (28.4%)	20 (39.2%)	0.123
Age, years	64.4 ± 10.9	64.4 ± 10.9	65.4 ± 11.3	0.522
Male sex, n (%)	8848 (67.9%)	7915 (67.9%)	36 (70.6%)	0.791
Body mass index (kg/m^2^)	24.5 ± 3.1	24.5 ± 3.1	24.6 ± 3.7	0.803
Risk factors, n (%)				
Hypertension	7049 (60.2%)	7014 (60.1%)	35 (68.6%)	0.275
Dyslipidemia	7555 (64.5%)	7524 (64.5%)	31 (60.8%)	0.683
Smoking	3285 (28.0%)	3270 (28.0%)	15 (29.4%)	0.951
Diabetes mellitus	4057 (34.6%)	4035 (34.6%)	22 (43.1%)	0.258
Chronic kidney disease	2432 (20.8%)	2421 (20.8%)	11 (21.6%)	1.000
Anemia	2921 (24.9%)	2903 (24.9%)	18 (35.3%)	0.121
Previous history, n (%)				
Peripheral arterial disease	1453 (12.4%)	1445 (12.4%)	8 (15.7%)	0.617
Congestive heart failure	880 (7.5%)	875 (7.5%)	5 (9.8%)	0.722
Previous MI	839 (7.2%)	835 (7.2%)	4 (7.8%)	1.000
Previous PCI	1568 (13.4%)	1559 (13.4%)	9 (17.6%)	0.490
Previous CABG	150 (1.3%)	150 (1.3%)	0 (0.0%)	0.848
Previous stroke	813 (6.9%)	806 (6.9%)	7 (13.7%)	0.102
Laboratory measurements				
VerifyNow PRU	217.8 ± 78.7	217.5 ± 78.7	268.3 ± 71.5	< 0.001
VerifyNow ARU	444.1 ± 69.4	444.0 ± 69.3	490.2 ± 81.9	0.001
LVEF (%)	58.8 ± 10.6	58.8 ± 10.6	54.9 ± 13.0	0.058
WBC, × 10^3^/mm^3^	7.9 ± 3.0	7.9 ± 3.0	8.6 ± 3.8	0.148
Hemoglobin, g/dL	13.6 ± 1.8	13.6 ± 1.8	13.2 ± 2.0	0.101
Platelet, × 10^3^/mm^3^	233.6 ± 72.4	233.6 ± 72.4	238.9 ± 76.7	0.602
Creatinine, mg/dL	1.1 ± 1.0	1.1 ± 1.0	1.5 ± 2.0	0.092
GFR, mL/min/1.73 m^2^ (MDRD)	78.7 ± 27.1	78.7 ± 27.1	69.4 ± 27.3	0.014
HbA1c, %	6.6 ± 1.4	6.6 ± 1.4	6.7 ± 1.3	0.482
Total cholesterol, mg/dL	174.0 ± 44.5	174.0 ± 44.4	180.0 ± 57.9	0.471
HDL-cholesterol, mg/dL	44.0 ± 12.8	44.0 ± 12.8	45.6 ± 13.5	0.395
LDL-cholesterol, mg/dL	106.8 ± 43.4	106.6 ± 40.2	148.3 ± 253.7	0.271
Triglyceride, mg/dL	143.2 ± 98.3	143.3 ± 98.4	126.5 ± 77.5	0.149

Continuous variables were expressed in mean ± SD or median (IQR) as indicated.

AMI, acute myocardial infarction; ARU, aspirin reaction unit; CABG, coronary artery bypass graft; DES, drug eluting stent; GFR, glomerular filtration rate; HbA1c, hemoglobin A1c; HDL, high density lipoprotein; LDL, low density lipoprotein; LVEF, left ventricular ejection fraction; MDRD, Modification of Diet in Renal Disease; MI, myocardial infarction; PCI, percutaneous coronary intervention; PRU, P2Y12 reaction unit; WBC, white blood cell.

The procedural and angiographic characteristics are summarized in Table 2. Multivessel disease was more common in the EST group (31 (60.8%) vs. 4500 (38.6%), *p* < 0.001), while ACC/AHA lesion type was not significantly different between the two groups (*p* = 0.515). PCI for the left main (LM) or the left anterior descending (LAD) was more common in the non-EST group (7304 (62.6%) vs. 23 (45.1%), *p* = 0.015). The number, length, and diameter of the implanted stents did not differ significantly.

**Table 2:** Procedural and angiographic characteristics of patients according to the occurrence of early stent thrombosis. (PTRG-PFT).

	Overall (n = 11,714)	Early stent thrombosis (−) (n = 11,663)	Early stent thrombosis (+) (n = 51)	*p* value
Angiographic feature				
ACC/AHA lesion, n (%)				0.515
A/B1 type	5238 (44.7%)	5218 (44.7%)	20 (39.2%)	
B2/C type	6476 (55.3%)	6445 (55.3%)	31 (60.8%)	
No. of diseased vessels, n (%)				< 0.001
One	7170 (61.2%)	7150 (61.3%)	20 (39.2%)	
Two	3039 (25.9%)	3026 (25.9%)	13 (25.5%)	
Three	1505 (12.8%)	1487 (12.7%)	18 (35.3%)	
Multivessel disease, n (%)	4531 (38.7%)	4500 (38.6%)	31 (60.8%)	0.002
Bifurcation lesion, n (%)	1363 (11.6%)	1361 (11.7%)	2 (3.9%)	0.133
Chronic total occlusion lesion, n (%)	821 (7.0%)	819 (7.0%)	2 (3.9%)	0.555
Procedural data				
Multivessel PCI, n (%)	4544 (38.8%)	4513 (38.7%)	31 (60.8%)	0.002
Treated lesions, n (%)				
LM	572 (4.9%)	570 (4.9%)	2 (3.9%)	1.000
LAD	6960 (59.4%)	6938 (59.5%)	22 (43.1%)	0.026
LCX	3434 (29.3%)	3410 (29.2%)	24 (47.1%)	0.008
RCA	4460 (38.1%)	4437 (38.0%)	23 (45.1%)	0.373
PCI for LM or LAD, n (%)	7327 (62.5%)	7304 (62.6%)	23 (45.1%)	0.015
Number of stents, n	1.6 ± 0.8	1.6 ± 0.8	1.8 ± 0.9	0.117
Stent length, mm	35.9 ± 22.5	35.8 ± 22.5	43.4 ± 28.1	0.060
Stent diameter, mm	3.0 ± 0.4	3.0 ± 0.4	3.0 ± 0.5	0.770
1st generation DES	944 (8.1%)	936 (8.0%)	8 (15.7%)	0.081
Concomitant medications, n (%)				
Aspirin	11,409 (97.4%)	11,361 (97.4%)	47 (94.1%)	0.302
Clopidogrel	11,714 (100.0%)	11,663 (100.0%)	51 (100.0%)	1.000
Cilostazol	1219 (10.4%)	1203 (10.3%)	16 (31.4%)	< 0.001
Statin	10,379 (88.6%)	10,333 (88.6%)	46 (90.2%)	0.890

Continuous variables were expressed in mean ± SD or median (IQR) as indicated.

ACC, American College of Cardiology; AHA, American Heart Association; DES, drug-eluting stent; LAD, left anterior descending artery; LCX, left circumflex artery; LM, left main artery; PCI, percutaneous coronary intervention; RCA, right coronary artery.

### Association between HPR and early ST risk

**Figure 2:**
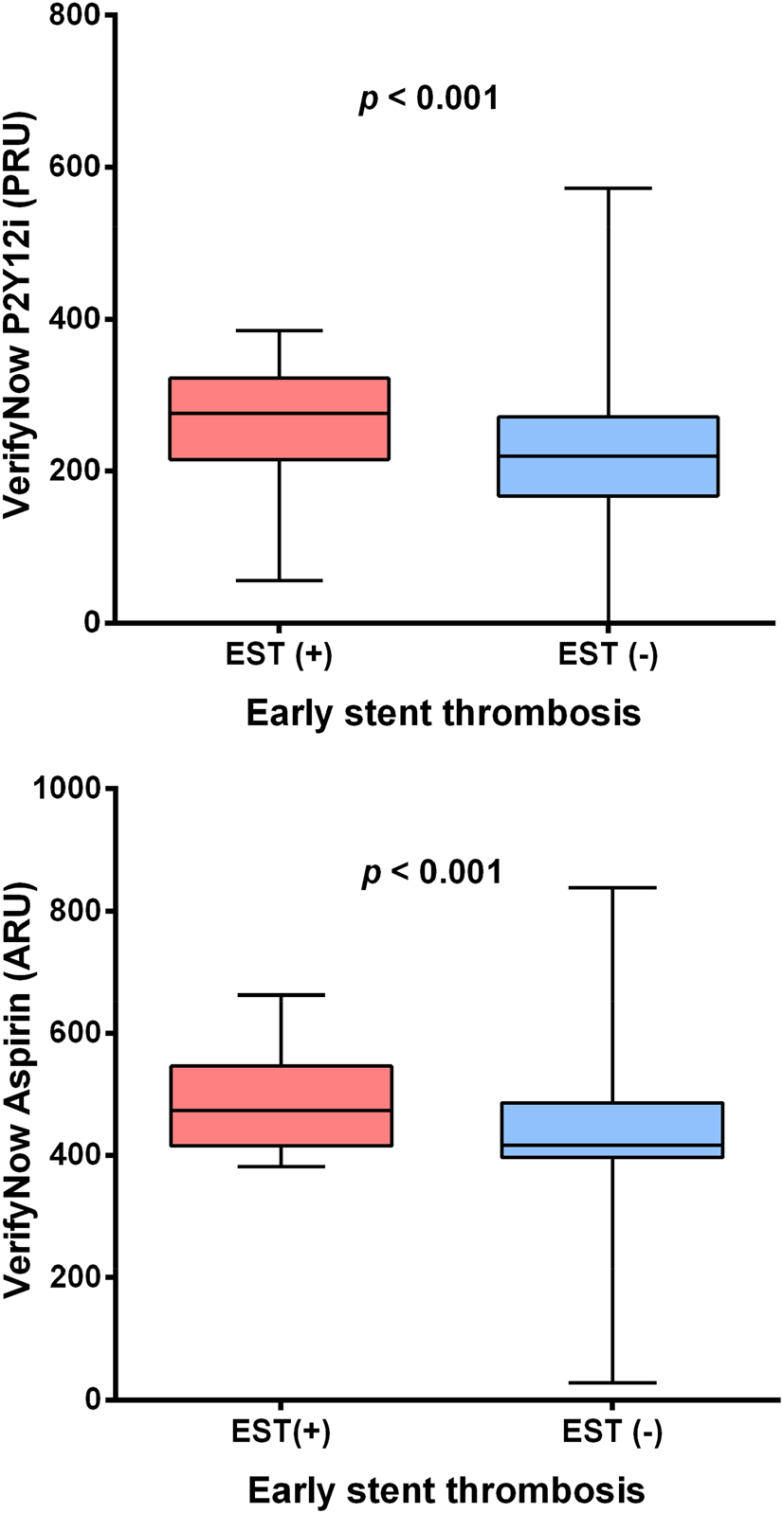
The PRU level by the occurrence of early stent thrombosis.

Patients who experienced EST had higher PRU values (268.3 ± 71.5 vs. 217.5 ± 78.2, *p* < 0.001) and higher ARU values (490.2 ± 81.9 vs. 444.0 ± 69.3, *p* = 0.001) compared to those without EST (Fig. 2). The EST group had a significantly higher proportion of patients with HPR, using either PRU ≥ 252 or ≥ 208 as described in previous literature (Supplemental Table S2)^7,9^.

In the multivariable analysis for the risk of EST occurrence (Table 3), having a PRU value of ≥ 252 was a significant predictor of EST (OR, 5.10; 95% CI 1.58–16.46; *p* = 0.006). Having an ARU value of ≥ 414 (OR 4.85; 95% CI 1.07–21.97; *p* = 0.040) was also associated with EST occurrence. The use of first-generation DES (OR 3.56; 95% CI 0.98–12.99; *p* = 0.054) also posed a higher risk for EST occurrence, but without statistical significance. Presentation as AMI did not significantly affect the risk of EST occurrence (OR 1.26; 95% CI 0.45–3.52; *p* = 0.660).

**Table 3:** Risk of early stent thrombosis.

Variables	Univariable OR (95% CI)	*p* value	Multivariable OR (95% CI)	*p* value
Women	0.88 [0.48; 1.61]	0.678	1.09 [0.41; 2.90]	0.869
Age ≥ 75	0.92 [0.45; 1.90]	0.831	0.18 [0.02; 1.42]	0.104
Body mass index (per kg/m^2^)	1.13 [0.65; 1.96]	0.667		
LVEF	0.97 [0.95; 0.99]	0.017	0.97 [0.93; 1.02]	0.227
Diabetes mellitus	1.43 [0.82; 2.50]	0.203	0.56 [0.19; 1.62]	0.284
Dyslipidemia	0.85 [0.49; 1.50]	0.579	0.64 [0.25; 1.65]	0.351
Hypertension	1.45 [0.80; 2.62]	0.219	2.59 [0.82; 8.16]	0.104
Peripheral artery disease	1.32 [0.62; 2.80]	0.477		
Chronic kidney disease	1.05 [0.54; 2.05]	0.887		
Congestive heart failure	1.34 [0.53; 3.38]	0.535		
Current smoker	1.07 [0.58; 1.96]	0.827		
Prior PCI	1.39 [0.67; 2.86]	0.373		
Anemia	1.65 [0.93; 2.93]	0.090	0.87 [0.30; 2.54]	0.797
Presentation as AMI	1.62 [0.92; 2.85]	0.092	1.26 [0.45; 3.52]	0.660
Multivessel diseases	2.47 [1.40; 4.33]	0.002	0.67 [0.21; 2.17]	0.505
Bifurcation lesion	0.31 [0.08; 1.27]	0.104	0.00 [0.00; 0.00]	0.990
CTO lesion	0.54 [0.13; 2.23]	0.394		
PCI at LM and/or LAD	0.49 [0.28; 0.85]	0.011	0.70 [0.27; 1.82]	0.468
1st generation DES	2.13 [1.00; 4.55]	0.050	3.56 [0.98; 12.99]	0.054
PRU ≥ 252	3.56 [2.00; 6.32]	< 0.001	5.10 [1.58; 16.46]	0.006
ARU ≥ 414	3.29 [1.22; 8.88]	0.019	4.85 [1.07; 21.97]	0.040

Univariable and multivariable analyses by logistic regression.

AMI, acute myocardial infarction; ARU, aspirin reaction unit; CTO, chronic total occlusion; DES, drug-eluting stent; LAD, left anterior descending artery; LVEF, left ventricular ejection fraction; LM, left main artery; PPI, proton pump inhibitor; PRU, P2Y12 reaction unit.

### Clinical outcomes after EST

**Figure 3:**
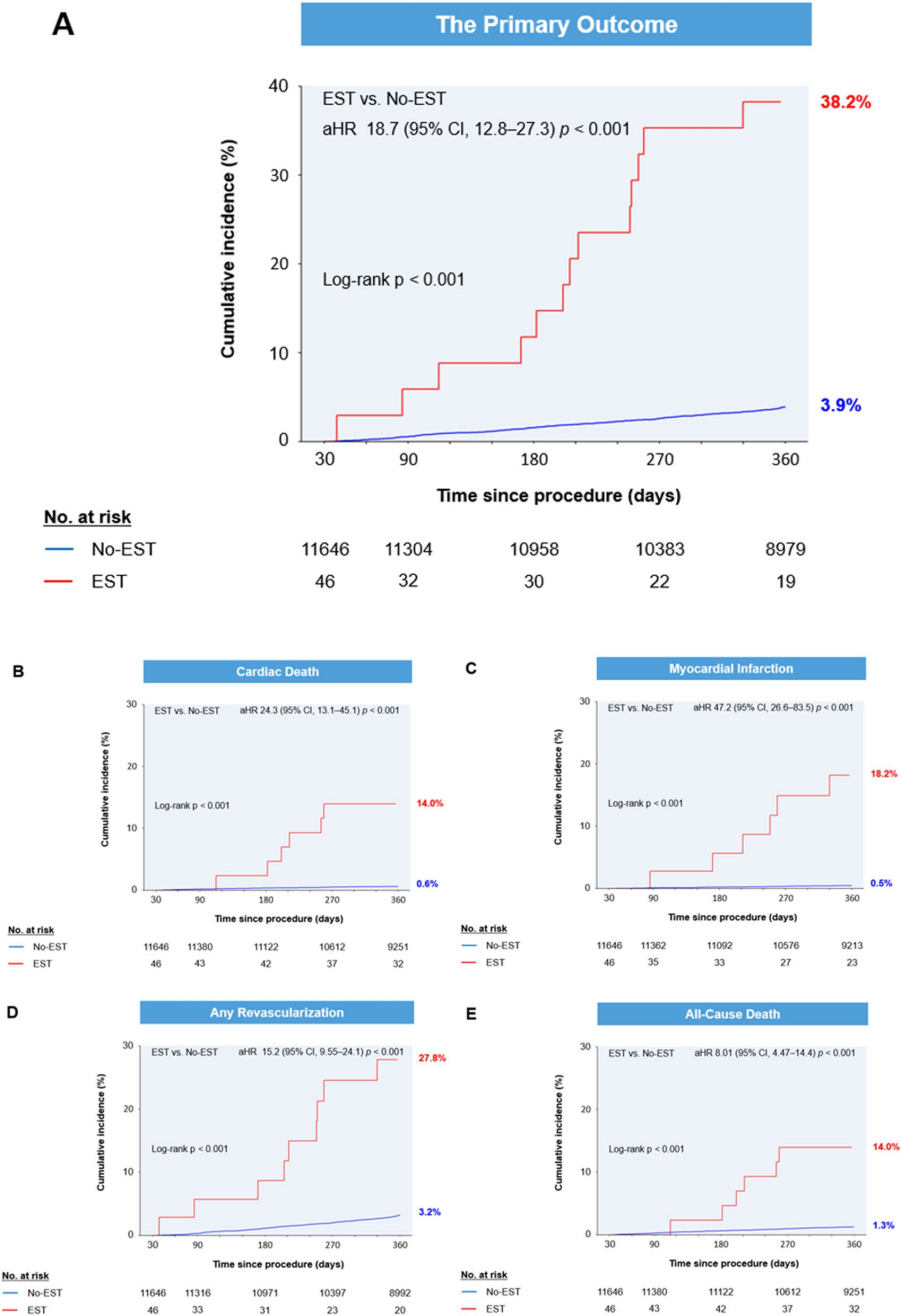
Kaplan–Meier curves for primary and secondary outcomes.

Out of the 11,714 patients in the study cohort, excluding the 22 patients who had died within 30 days after index PCI, 1-year follow-up for clinical outcomes was done for the remaining 11,692 patients (Fig. 1). The median duration overall of the entire follow-up period was 541 days (inter-quartile range; 365–1750 days). The cumulative incidence of the primary composite outcome (cardiac death, MI, any revascularization) at 1 year was higher in the EST group (38.2% vs. 3.9%, Log-rank *p* < 0.001) (Table 4 and Fig. 3). The secondary outcomes of all-cause death (14.0% vs. 1.3%, Log-rank *p* < 0.001), cardiac death (14.0% vs. 0.6%, Log-rank *p* < 0.001), MI (18.2% vs. 0.5%, Log-rank *p* < 0.001) and any revascularization (27.8% vs. 3.2%, Log-rank *p* < 0.001) were also significantly higher in the EST group.

**Table 4:** Cumulative incidence of outcomes at 1 year.

	Overall cohort			
	Overall (n = 11,692)	Early stent thrombosis (−) (n = 11,646)	Early stent thrombosis (+) (n = 46)	Log-rank *p*
Primary outcome				
Composite of cardiac death, myocardial infarction, and any revascularization	435 (4.0%)	422 (3.9%)	13 (38.2%)	< 0.001
Secondary outcomes				
All-cause death	144 (1.3%)	138 (1.3%)	6 (14.0%)	< 0.001
Cardiac death	71 (0.6%)	65 (0.6%)	6 (14.0%)	< 0.001
Myocardial infarction	56 (0.5%)	50 (0.5%)	6 (18.2%)	< 0.001
Any revascularization	353 (3.3%)	344 (3.2%)	9 (27.8%)	< 0.001

Values are presented as numbers (the Kaplan–Meier estimate of cumulative incidence). CI, confidence interval; HR, hazard ratio.

## Discussion

From this multicenter registry of patients with DES implantation, the main findings of our study are as follows: (1) ST is not rare even in the 2nd generation DES era, and most of the ST events occurred within the first 30 days (early ST); (2) patients with EST had a higher PRU, and a higher PRU value was an independent predictor of EST; (3) even in the 2nd generation DES era, the occurrence of EST led to a strikingly high incidence of subsequent cardiovascular events during the 1-year follow-up.

Despite advancements in DES and PCI techniques, the prevalence of ST remains high. The prevalence of EST sits at around 0.5–1.0% in real-world registries, depending on the stent types and clinical presentations^2,10,11^. In a meta-analysis of 30 studies including 221,066 patients, acute and subacute ST events were estimated to be 0.4% and 1.0%, respectively^12^. This is in accordance with our results, whereby EST occurred in 0.4% of the total population. Extensive research in the field of ST, however, have declined since its peak spotlight during the transition from bare-metal stents (BMS) to DES. In a recent report by Batchelor et al. on stent thrombosis in contemporary PCI, in 41,137 consecutive patients with PCI procedure, patients with EST tended to be female, diabetic and have reduced left ventricular ejection fraction^2^. EST was also associated with increased short-term mortality at 30 days (23.6% vs. 2.0, *p* < 0.001)^2^. The ADAPT-DES registry of 8,582 patients undergoing PCI showed that high platelet reactivity (HPR) in patients presenting with ACS posed a high risk of EST^11^. Notably, after 30 days, relative ST risks were not significantly different amongst different clinical presentation groups (ACS vs. non-ACS)^11^. Long-term outcomes after EST were assessed by Ishihara et al., in which 187 cases of definite ST were evaluated^3^. Cumulative mortality after a median follow-up of 1054 days reached 14.6% at 1 year and 33.8% at 10 years^3^. Long-term outcomes after EST in relation to HPR, however, have not been fully elucidated in previous literature; to the best of our knowledge, our study is the first to do so.

Several investigations have previously suggested that ST can occur in patients on dual antiplatelet therapy due to reduced platelet inhibition by aspirin and thienopyridines^13,14^. Clopidogrel shows variability in clinical efficacy, owing to being a prodrug that requires metabolic activation in the liver. Interruption of full platelet inhibition by clopidogrel is a risk factor for EST. In a case–control study of patients with definite EST, a reduced CYP2C19 metabolic status (adjusted OR 1.99), use of PPI (adjusted OR 2.19) and higher clopidogrel loading doses (adjusted OR 0.73) were associated with EST^15^. A collaborative analysis of 17 studies and 20,839 patients yielded a 2.7-fold increased risk for ST in patients with HPR, defined as > 208 PRU for VerifyNow^16^. Notably, in an analysis of stent thrombosis from early to very late stages, a higher clopidogrel loading dose was associated with a lower risk of EST but not other ST timings^17^. Our study from the PTRG-DES registry reports a fivefold increased risk of EST by high on-treatment PRU values in patients undergoing DES-PCI. A high PRU value (≥ 252) was the strongest predictor of EST in multivariable analysis, followed by a high ARU value (≥ 414). At 30 days, all patients were on dual antiplatelet therapy which included clopidogrel, giving weight to between-individual PRU differences acting as a driving factor for EST. Likewise, based on the ADAPT-DES registry, Stone et al. have previously reported that increased HPR on clopidogrel is associated with elevated ST risk, especially in patients presenting with ACS^11^. The landmark analyses showed that the increased risk by HPR was predominant in the first 30 days after PCI, consistent with the notion that most ST cases are also EST cases. It does not, however, evaluate the long-term cardiovascular events after an ST event has taken place.

Additionally, our results support the usage of a different cut-off value for East Asian populations as suggested by previous studies^18,19^. While the expert consensus statement on platelet reactivity suggests the cut-off value of > 208 PRU, the main trials that prompted this value were the TRIGGER-PCI and the ANTARCTIC studies, both of which were carried out in sites with primarily Caucasian populations^20–22^. Furthermore, neither study has specified the ethnic background of the study participants. One the other hand, studies based on populations with East Asian background showed that ≥ 252 was the optimal cut-off value for PRU. A sensitivity analysis using > 208 as the cut-off value showed similar tendencies, but ≥ 252 yielded better predictive values with a higher hazard ratio than > 208 (Supplemental Table S2).

In our study, while HPR was associated with EST occurrence, one-year cardiovascular outcomes were worse for patients in whom EST had occurred. This was consistent across the primary and secondary outcomes of all-cause death, cardiac death, MI and any revascularization. In a paper by Lee et al. on the PTRG-DES registry, HPR was associated with increased mortality and adverse cardiovascular outcomes at 1 year and at 5 years^9^. Among secondary outcomes studied, ST occurred in 0.9% of the high PRU group, which was significantly higher than intermediate or low PRU groups (*p* < 0.001). Although ST was not a component of the primary composite outcome of major adverse cardiac and cardiovascular events (MACCE), considering the hierarchy of events, it appears likely that ST influenced the subsequent occurrence of cardiovascular events such as mortality or MI^9^. Recently, Ishihara et al. reported the 10-year cumulative mortality after ST occurrence to be over 33%^3^. Multivariable analysis including clinical diagnoses, target vessel, ST timing, clinical presentation and initial/final TIMI flow grade showed DM, hemodialysis, target lesion in left anterior descending artery or left main trunk and late ST to be independent predictors of mortality. Platelet reactivity, however, was not included in their analysis; in our study, after adjusting with PRU values, the above variables lost significance in multivariable analysis.

The risk posed by 1st generation DES usage (vs. 2nd generation) was increased for EST occurrence in our study, but without statistical significance (HR 3.56, 95% CI 0.98–12.99, *p* = 0.054). Further, in a sensitivity analysis for patients with 2nd generation stent implantation, HPR was an independent predictor of EST with an adjusted OR of 3.99 (Supplemental Table S3). Understandably, stent types were frequently associated with stent thrombosis risks in previous studies. Most of the results, however, were from comparisons between BMS and DES rather than amongst DES generations^23–25^. Some of the early studies even suggested that 1st generation DES promoted stent thrombosis compared with BMS^10,24,25^. The increased risk attributable to 1st generation DES, however, was mainly noticeable for late or very late stent thromboses, with little difference in EST occurrence^10,26^. For EST occurrence, therefore, it appears likely that although 2nd generation DES may be associated with decreased risks, future studies with larger sample sizes are needed to further extrapolate our findings. However, as the earlier generations of intracoronary stents are increasingly out of use, the clinical value of such studies may need careful reasoning.

## Limitations

Our study has several limitations. First, it was a retrospective, observational study; thus, the possibility of selection bias and unmeasured confounding factors cannot be excluded. As only the patients with clopidogrel usage were enrolled, a possible selection bias in patients with acute coronary syndrome with other potent P2Y12 inhibitors remains; patients who were prescribed with the more potent P2Y12 inhibitors, either before or after enrollment, were excluded from the registry. However, although it was a retrospective cohort study, the multicenter nature of the cohort allowed the sample size to be relatively large compared with other previous studies. Second, being an exclusively all-Korean registry, the results and interpretations of our study should be applied with caution in patients with other ethnic backgrounds. This is especially relevant considering the well-known East Asian paradox, with higher reported prevalence of bleeding in East Asian populations^27^. The higher PRU associated with EST and adverse outcomes in our study suggests that other potent P2Y12 inhibitors should be considered in patients with HPR. Considering the bleeding risks after PCI, some eastern Asian physicians may opt for the less potent clopidogrel over other P2Y12 inhibitors, which should be done carefully in patients with HPR. Third, specific loading strategies for clopidogrel were not available for analysis, but all patients were loaded adequately with one of the strategies as described in the methods section. Fourth, the PRU levels were obtained only within 24 h of the index PCI, as described in the methods section. Therefore, data pertaining to the PRU levels at the time of clinical events or during the follow-up were not available. Fifth, details regarding intravascular ultrasound (IVUS) or other intravascular imaging modalities during PCI were not available. Sixth, treatment adherence before the index date was not available.

## Conclusions

HPR was associated with a higher incidence of early stent thrombosis and adverse cardiovascular events after EST occurrence in patients undergoing coronary DES implantation. Evaluation of platelet reactivity should therefore be considered in patients on clopidogrel after PCI to reduce the incidence of EST and the adverse outcomes thereupon.

### Supplementary Information

Supplementary Information.

## Data Availability

The data used to support the findings of this study are available from the corresponding author upon reasonable request.

## Abbreviations

ARU: Aspirin reaction unit
DES: Drug-eluting stent
HPR: High platelet reactivity
PCI: Percutaneous coronary intervention
PFT: Platelet function test
PRU: P2Y12 inhibitor reaction unit
PTRG-DES: Platelet function and genoType-Related long-term proGnosis in DES-treated patients
