# Supplementary material for: High platelet reactivity strongly predicts early stent thrombosis in patients with drug-eluting stent implantation
Source: Sci Rep. 2024 Jan 4;14:520. doi: 10.1038/s41598-023-50920-9 (PMC10766995; doi:10.1038/s41598-023-50920-9)
Supplement: Supplementary file 1 — Supplementary Information. [file 41598_2023_50920_MOESM1_ESM.docx]

**Supplemental table S1. CY2C19 genotypic status according to EST status**

|  | **EST (N=25)** | **No EST (N=5,792)** | **P-value** |
| --- | --- | --- | --- |
| CYP2C19 status |  |  | 0.006 |
| Extensive metabolizer | 3 (12.0%) | 2526 (37.7%) |  |
| Intermediate metabolizer | 14 (56%) | 2317 (48.1%) |  |
| Poor metabolizer | 8 (32.0%) | 949 (14.2%) |  |

EST = early stent thrombosis

**Supplemental table S2. Relationship between platelet reactivity and EST**

|  | **EST (N=51)** | **No EST (N=11,663)** | **P-value** |
| --- | --- | --- | --- |
| PRU ≥208 | 40 (78.4%) | 6507 (55.8%) | 0.002 |
| PRU ≥252 | 33 (64.7%) | 3968 (34.0%) | <0.001 |

EST = early stent thrombosis; PRU = P2Y12 reactivity unit.

**Supplemental table S3. Risk of early stent thrombosis in patients with second-generation DES implantation**

| **Variables** | **Univariable**  **OR (95% CI)** | **p-value** | **Multivariable**  **OR (95% CI)** | **p-value** |
| --- | --- | --- | --- | --- |
| Women | 0.79 [0.43;1.46] | 0.447 | 0.96 [0.40;2.32] | 0.926 |
| Age ≥75 | 1.01 [0.50;2.02] | 0.981 | 0.17 [0.02;1.30 | 0.087 |
| Body mass index (per kg/m^2^) | 0.33 [0.77;2.30] | 0.312 |  |  |
| LVEF | 0.98 [0.95;1.00] | 0.060 |  |  |
| Diabetes mellitus | 1.44 [0.82;2.50] | 0.202 | 0.90 [0.37;2.16] | 0.808 |
| Dyslipidemia | 0.83 [0.47;1.46] | 0.526 | 0.93 [0.39;2.21] | 0.865 |
| Hypertension | 1.60 [0.88;2.93] | 0.126 | 1.71 [0.66;4.42] | 0.270 |
| Peripheral artery disease | 1.45 [0.70;2.99] | 0.312 |  |  |
| Chronic kidney disease | 1.19 [0.62;2.28] | 0.592 |  |  |
| Congestive heart failure | 1.35 [0.54;3.42] | 0.522 |  |  |
| Current smoker | 1.15 [0.64;2.08] | 0.643 |  |  |
| Prior PCI | 1.57 [0.79;3.15] | 0.201 |  |  |
| Anemia | 1.80 [1.02;3.18] | 0.043 | 0.89 [0.34;2.33] | 0.809 |
| Presentation as AMI | 1.71 [0.98;2.99] | 0.060 |  |  |
| Multivessel diseases | 2.64 [1.50;4.67] | 0.001 | 1.05 [0.40;2.75] | 0.917 |
| Bifurcation lesion | 0.49 [0.15;1.56] | 0.225 |  |  |
| CTO lesion | 0.54 [0.13;2.23] | 0.3972 |  |  |
| PCI at LM and/or LAD | 0.48 [0.28;0.84] | 0.010 | 0.58 [0.25;1.35] | 0.204 |
| PRU ≥252 | 3.37 [1.91;5.95] | <0.001 | 3.99 [1.54;10.32] | 0.004 |
| ARU ≥414 | 3.28 [1.21;8.89] | 0.020 | 2.35 [0.84;6.58] | 0.104 |

Univariable and multivariable analyses by logistic regression.

AMI= acute myocardial infarction; ARU = aspirin reaction unit; CTO = chronic total occlusion; DES= drug-eluting stent; LAD = left anterior descending artery; LVEF= left ventricular ejection fraction; LM = left main artery; PPI = proton pump inhibitor; PRU = P2Y12 reaction unit.
